# Erysipelas—Its Rapid Cure

**Published:** 1885-11

**Authors:** Daniel Lewis


### Erysipelas—Its Rapid Cure.

While several American surgeons have mentioned lead paint in these cases, the credit of bringing it prominently before the profession is due to Mr. Richard Barwell, of Charing Cross Hospital, who, in the *Lancet* of March 10, 1883, described what he termed "A rapidly successful treatment of erysipelas," which consisted in painting the parts thoroughly with white lead paint, dressing the wound, if there be any, by cotton wool saturated with boro-glyceride. The effect was remarkably and quickly successful; cases after operations on the arm for necrosis, and other hospital cases, being well in a few days. The pain was relieved almost at once, and only such after-application needed as to keep the coating perfect. In idiopathic erysipelas, he found it equally successful.

Since the publication of Mr. Barwell's cases, I have used no other local application for erysipelas, and have often used no internal treatment except the purge as he recommends.

Pure white lead paint of the shops is likely to dry too slowly, and I tell the painter to add some drier, as in ordinary painting, which in no way changes the effect of the application.

I am unable to give the composition of this drier, as it is a patent preparation; but painters tell me it is some kind of resin dissolved in linseed oil.

The paint should be thicker than for ordinary use. It peels off readily when desquamation begins, even from the head, where I have often applied it.

The mention of cases in detail seems unnecessary, but several instances of especial interest have been noted. A man whose right ear was completely involved was relieved at once of the burning pain, and recovered without a second application. The same rapid results have been obtained in my practice when the disease involved the nose, face, and various other parts of the body.

Being hastily summoned to a patient who was attacked with facial erysipelas, I found that the disease began thirty-six hours previously, and had rapidly spread over the entire face. The temperature was 103 deg. in the axilla, and the pain was severe. The husband was a painter, and had the white lead paint and drier in the house. It was thoroughly applied over the face, and they were requested to report the condition of the patient on the following day. They failed to do so, and months afterward, when visiting a patient in the same family, I learned that the single application cured the disease.

Mr. Barwell reports similar rapid results in traumatic cases, and even in hospital cases.

While the application of this remedy gives the patient a somewhat striking appearance at times, when a single ear or the nose is affected, for example, yet it is a dry and cleanly dressing, very easily applied, and as successful as can be desired. It is, beyond all question, preferable to any means which have been in use up to the present time, and is still entitled to its designation as a rapidly successful treatment of erysipelas.—*Dr. Daniel Lewis, Jour. C. and V. Diseases.*

